# Oncofertility: Pharmacological Protection and Immature Testicular Tissue (ITT)-Based Strategies for Prepubertal and Adolescent Male Cancer Patients

**DOI:** 10.3390/ijms20205223

**Published:** 2019-10-21

**Authors:** Elissavet Ntemou, Chrysanthi Alexandri, Pascale Lybaert, Ellen Goossens, Isabelle Demeestere

**Affiliations:** 1 Research Laboratory in Human Reproduction, Faculty of Medicine, Université Libre de Bruxelles (ULB), B-1070 Brussels, Belgium; Chrysanthi.Alexandri@ulb.ac.be (C.A.); idemeest@ulb.ac.be (I.D.); 2 Laboratory of Physiology and Pharmacology, Faculty of Medicine, Université Libre de Bruxelles (ULB), B-1070 Brussels, Belgium; plybaert@ulb.ac.be; 3 Biology of the Testis (BITE) Laboratory, Department of Reproduction, Genetics and Regenerative Medicine, Vrije Universiteit Brussel (VUB), 1090 Brussels, Belgium; ellen.goossens@vub.ac.be

**Keywords:** testis, prepubertal boys, adolescent males, cancer, gonadotoxic treatment, pharmacological protection, ITT, fertility preservation, restoration, oncofertility

## Abstract

While the incidence of cancer in children and adolescents has significantly increased over the last decades, improvements made in the field of cancer therapy have led to an increased life expectancy for childhood cancer survivors. However, the gonadotoxic effect of the treatments may lead to infertility. Although semen cryopreservation represents the most efficient and safe fertility preservation method for males producing sperm, it is not feasible for prepubertal boys. The development of an effective strategy based on the pharmacological protection of the germ cells and testicular function during gonadotoxic exposure is a non-invasive preventive approach that prepubertal boys could benefit from. However, the progress in this field is slow. Currently, cryopreservation of immature testicular tissue (ITT) containing spermatogonial stem cells is offered to prepubertal boys as an experimental fertility preservation strategy by a number of medical centers. Several in vitro and in vivo fertility restoration approaches based on the use of ITT have been developed so far with autotransplantation of ITT appearing more promising. In this review, we discuss the pharmacological approaches for fertility protection in prepubertal and adolescent boys and the fertility restoration approaches developed on the utilization of ITT.

## 1. Introduction: The Quest for Fertility Preservation and Restoration

The prevalence of cancer in children and adolescents (0–19 years of age) has increased by 27% over the last decades, while the likelihood to be diagnosed with cancer is higher in boys than in girls [1]. The most commonly diagnosed types of cancer in prepubertal boys and adolescents are leukemia, brain and other central nervous system tumors, and lymphomas, whereas testicular germ cell tumors are frequently observed in adolescents [2]. For these patients, individual modern cancer treatment approaches can be lifesaving as indicated from the high (more than 80%) 5-year survival rate for childhood cancer patients [3,4,5]. Nonetheless, exposure to chemotherapeutic regimens and/or radiation may severely affect their reproductive ability in the long-term. It is estimated that almost half of male childhood cancer survivors will experience difficulties to conceive a child during adulthood which presents a significant quality-of-life issue [6,7,8].

Oncofertility, a term introduced by Woodruff in 2007 [9], is an emerging cross-disciplinary field that involves a variety of fertility preservation strategies for patients diagnosed with cancer and who are at risk of becoming infertile due to the gonadotoxicity of the treatment. However, fertility preservation is not solely offered to cancer patients but also to patients facing other fertility-compromising therapies including conditioning regimen prior to bone marrow transplantation for benign diseases [10].

Although cancer treatment protocols have been amended to promote health and minimize the side effects [11], there is still an immense amount of work to be done in the field of fertility preservation for boys and adolescents. Before any invasive procedure, the development of preventive approaches aiming at the protection of germ cells and testicular function during the exposure are preferred. Moreover, the implementation of such approaches would enable natural conception to occur. However, due to the slow progress up to now in this field, alternative strategies have been developed based on the use of cryopreserved immature testicular tissue (ITT) collected from the boys preferably before the start of the treatment. At the moment, at least 16 centers are offering this service worldwide with 7 of them in Europe [10,12,13,14].

In the first part of this paper we review the current approaches on male gonadal protection and introduce a new promising approach based on the use of microRNAs (miRNAs), while in the second part we review the in vitro and in vivo experimental strategies developed based on the use of ITT.

## 2. Testicular Development and Function

The testis is a unique specialized organ with two main functions: the production of gametes and androgens. Each testis contains a number of convoluted seminiferous tubules and it is structurally and functionally divided into the intratubular and the interstitial compartment. The first compartment consists of the germ cells, Sertoli cells (which are the nourishing cells in contact with germ cells and closely attached to the basement membrane) and peritubular myoid cells (which secrete several factors and surround the tubules). The components of the second compartment are the Leydig cells (whose main function is to secrete androgens), blood vessels, immune cells and connective tissue. The continuous sperm production depends on the capacity of spermatogonial stem cells (SSCs), a subpopulation of spermatogonia, to self-renew and to differentiate into more advanced germ cell types under hormonal regulation [15,16,17].

**Figure 1:**
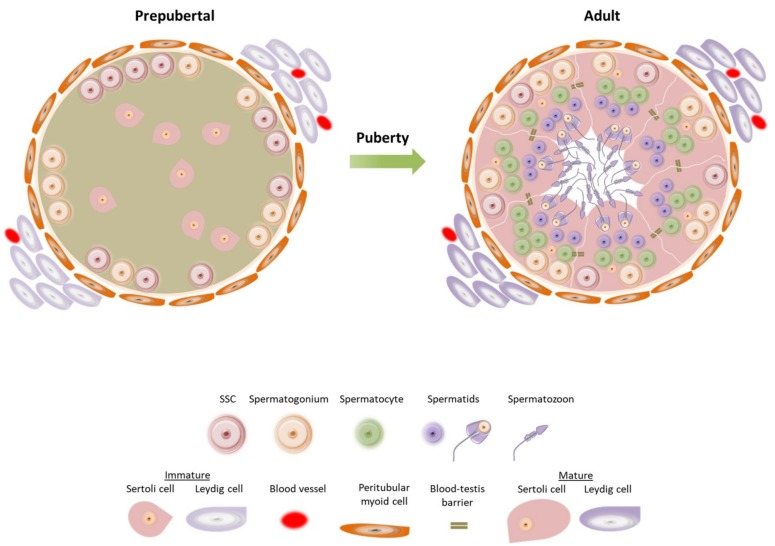
Schematic representation of a seminiferous tubule cross-section from a prepubertal and adult testis. The seminiferous tubule of a prepubertal testis is surrounded by immature peritubular myoid cells and immature Leydig cells in the interstitial tissue. Spermatogonial stem cells (SSCs) and spermatogonia reside on the basement membrane and immature proliferative Sertoli cells are in the center of the tubule. At that point, Sertoli cells strongly express anti-Müllerian hormone (AMH) while androgen receptor (AR) is absent. Before the end of prepuberty and during puberty the expression of AMH is gradually reduced until adulthood when Sertoli cells are fully matured, AR is present and AMH is no longer detectable. The seminiferous tubule of an adult testis is surrounded by mature peritubular myoid cells and mature Leydig cells and it contains germ cells at different developmental stages.

**Figure 2:**
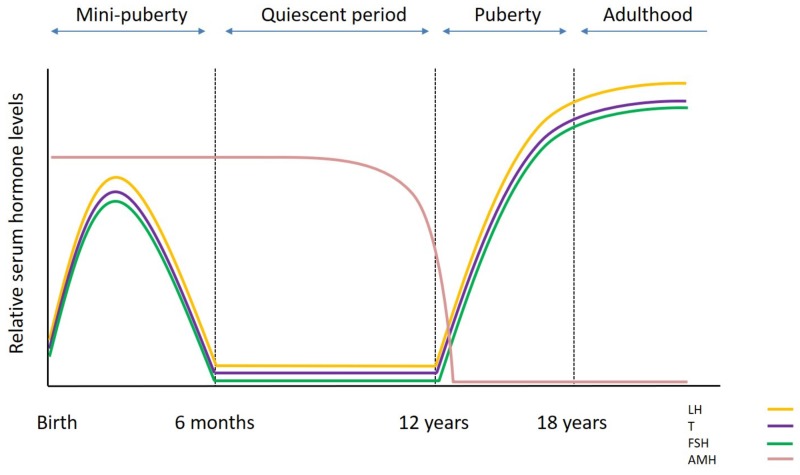
Hormonal levels in males from birth till adulthood.

The prepubertal testis differs from the adult testis as it goes through a number of cellular modifications driven by changes of the endocrine environment from birth through puberty [18] (Figure 1). Just after birth the gonocytes continue to proliferate till 6 months of age and then they differentiate into A_dark_ spermatogonia which are proposed to be the “true” SSCs [19,20]. Spermatogonia/SSCs is the only germ cell population present during prepubertal life until the onset of spermatogenesis at puberty. During the first year of age the prepubertal testis contains immature proliferative Sertoli cells; fetal Leydig cells are present up to 6 months post-natal, and then they are replaced by adult Leydig cell precursors (immature Leydig cells). The levels of gonadotrophins and testosterone (T) increase following birth (mini-puberty) until the “quiescent” period where the levels drop. In particular, follicle stimulating hormone (FSH) and luteinizing hormone (LH) release reaches a peak at 4–10 weeks post-natal, before falling to the lowest levels at around 6 months. Similarly, T production from Leydig cells reaches the highest levels at around the third month before dropping to prepubertal levels at 6–9 months [20] (Figure 2). The absence of androgen receptor (AR) expression in immature Sertoli cells does not allow spermatogenesis to progress further during that period, while the production of anti-Müllerian hormone (AMH) remains high.

Around puberty, the pulsatile secretion of gonadotrophin-releasing hormone (GnRH) stimulates a progressive surge in gonadotrophin release with FSH promoting the proliferation of immature Sertoli cells and LH inducing the maturation of Leydig cells (adult Leydig cells), which start to produce T again [20,21]. The increase of T levels stimulates Sertoli cell maturation, which now express AR and are not able to mitotically divide any further, and inhibits AMH expression [21]. The lumen starts to expand and a layer of mature peritubular myoid cells separates the seminiferous tubules from the interstitial compartment. The transformation of the testis from a prepubertal to postpubertal status is completed with the development of tight junctions between the Sertoli cells and the formation of the blood–testis barrier [22]. The blood–testis barrier is essential for the protection of the haploid germ cells from the immune system. Ultimately, germ cells can enter meiosis and complete their differentiation into haploid cells, the spermatozoa, with a direction from the basement membrane towards the lumen [18].

## 3. Cancer Treatment and Fertility Impairment

In contrast to previous knowledge [23], the prepubertal testis appears to be more sensitive to oncological treatments than the adult testis as the testicular environment is not quiescent but there is a constant turnover of early germ cells [24,25]. Establishment of spermatogenesis during puberty will depend on the degree of damage caused by the treatment either direct to SSCs or indirect through Sertoli and Leydig cell impairment. Complete depletion of SSCs is translated to permanent azoospermia [26,27].

The exact cytotoxic effect of the different chemotherapeutic agents on the testicular germinal epithelium and the risk of impaired fertility cannot be easily assessed, as they are subject to the dose and duration of treatment, the age of the patient and his sensitivity [28,29]. Chemotherapeutic agents are categorized into high, moderate or low risk of impaired fertility. The high-risk category includes alkylating (e.g., cyclophosphamide, cisplatin, busulfan, procarbazine and ifosfamide) and platinum (e.g., cisplatin) agents which act by direct DNA and RNA damage and the activation of apoptotic pathways. Treatment of prepubertal boys with high cumulative doses of cyclophosphamide can induce testicular damage, depletion of SSCs and reduction of tubular fertility index [30,31,32]. Moreover, a study from Chow et al., (2016) which included more than 10,000 childhood cancer survivors (both males and females) diagnosed before 21 years of age and not exposed to gonadal or brain radiation, demonstrated that male survivors, especially those exposed to high cumulative doses of alkylating drugs and cisplatin, were less likely to achieve a pregnancy or a live birth compared to their siblings [33]. The second category, associated with moderate or low risk, includes the antimetabolites (e.g., methotrexate) whose mechanism of action relies on the inhibition of DNA and RNA synthesis and inhibition of mitosis [25,33].

The testis is also sensitive to radiation exposure and the potential damage depends on the total dose, treatment field and fractionation [34]. Doses as low as 0.1 Gy can impair spermatogenesis for a short time while doses of 2–3 Gy can cause long-term azoospermia. Higher doses of 4–6 Gy can deplete SSCs resulting in profound impairment of spermatogenesis [35,36]. Studies demonstrated that total body irradiation with more than 7.5 Gy reduces the likelihood of male childhood cancer survivors to achieve pregnancy while no pregnancies were reported following more than 10 Gy for childhood acute lymphoblastic leukemia [6,37]. Additionally, cranial irradiation with 24 Gy is also associated with reduced fertility related to hormonal dysfunction [38].

In the case of gonadotoxic (chemotherapy and radiation) exposure of the testes with ongoing spermatogenesis (adolescent or adult), a significant reduction of sperm concentration within 2 months is observed [39]. Spontaneous recovery of spermatogenesis is possible even 5 years after the treatment [40]. However, re-establishment of spermatogenesis, similar to prepubertal testis, depends on the cytotoxic effect of the treatment as depletion of spermatogonia, including SSCs, and/or morphological damage or dysfunction of the supporting Sertoli cells, would result in permanent azoospermia.

## 4. Strategy Based on Cryopreservation of Sperm

The first-line strategy in fertility preservation in males is sperm cryopreservation. For adolescent and adult males, sperm banking represents a relatively easy, safe and clinically approved option to safeguard fertility [10]. Long-term stored ejaculated, or testicular, spermatozoa followed by thawing at the time of child wish, can be used to achieve pregnancy by assisted reproductive techniques. It is recommended that semen samples should be collected before the onset of gonadotoxic treatment as sperm quality and DNA integrity may be compromised [41]. However, sperm quality is also known to be impaired in patients with malignancies even before treatment [41,42,43] and in addition, adolescents engaged in sperm banking often face difficulties in producing semen samples [44,45]. Moreover, the lack of sperm production before the age of 13 to 14 years renders this approach not applicable for prepubertal boys and urges the development of other methods.

## 5. Experimental Strategies Based on Pharmacological Protection of the Testis

The pharmacological protection of the testis during oncological treatments is a new emerging field in male oncofertility and appears to be very attractive, especially for prepubertal boys who cannot benefit from standard semen cryopreservation. The administration of pharmacological agents concomitantly to chemotherapy in order to prevent damage of germ cells and testicular somatic cells and to maintain the testicular function could become a non-invasive preventive approach to preserve fertility.

### 5.1. Hormonal Protection (GnRHa)

Currently, there is no clinically approved pharmacological option to protect spermatogenesis during gonadotoxic treatments, but studies are ongoing at the preclinical level. More specifically, the temporary suppression of gonadotrophins using GnRH agonists or antagonists (GnRHa) has been tested in adult rodents and non-human primates with controversial results. According to these studies, the direct or secondary hormonal suppression in rats exposed to chemotherapeutic agents like procarbazine or busulfan could restore fertility and promote the spermatogenic resumption [46,47], while in the mouse model no protective effect was detected [48]. In other animal species, like dogs or monkeys, the hormonal protection of the testicular function did not result in an increased reproductive outcome [49]. However, a study of Shetty et al. (2013) revealed that the transient suppression of gonadotrophins’ function could enhance the endogenous spermatogenic recovery in cynomolgus monkeys that were exposed to radiation, in a dosage similar to radiotherapy in cancer patients [50]. It has been shown that following irradiation both intratesticular T and FSH increase and hence, it can be hypothesized that their inhibition may contribute to maintenance of normal spermatogenesis [51]. It is not clear yet how the increased levels of intratesticular T or FSH are able to inhibit spermatogenesis during oncological treatments, but it seems that the maintenance of peripheral T is important for supporting the male functions. In general, the knowledge about the mechanism of GnRHa action on gonadal protection is limited, but there are some evidence-based scenarios trying to elucidate it [49]. According to these, highly dividing cells are more vulnerable to cytotoxic agents and therefore, administration of GnRHa during oncological treatments can slow down the proliferation rates leading to gonadal quiescence and protection of cells from apoptosis. In this way, suppression of the hypothalamic–pituitary–gonadal axis leads to reduced spermatogenesis and renders the testes more resistant to chemotherapy. Nevertheless, the application of these pharmacological agents in humans is not recommended based on the outcome of the clinical trials. More specifically, from the seven clinical trials on hormonal-based therapies only one provided protection to spermatogenesis against gonadotoxic therapies [49]. However, the small cohort of patients and controversies regarding the study design in these trials make it difficult to reach definite conclusions. Moreover, the controversial results about the effectiveness of GnRH analogues may be attributed to the differences in hormonal and spermatogenetic regulation between the species [52]. In rats, mainly T is required for spermatogenesis while in the cynomolgus monkeys and humans, T, LH and FSH act together to stimulate normal spermatogenesis [53]. Besides, in prepubertal patients, administration of GnRHa would not provide any protection as the hypothalamic–pituitary–gonadal axis is not active [54]. Consequently, the identification of the underlying mechanisms involved in androgen inhibition during cytotoxic treatments will help to develop new and more efficient agents for humans while better designed clinical trials can validate the previous observations.

### 5.2. Granulocyte Colony-Stimulating Factor (G-CSF)

The granulocyte colony-stimulating factor (G-CSF) is administered to cancer patients in order to address neutropenia, a common hematopoietic side-effect during oncological treatments. However, preclinical studies in a mouse model revealed that G-CSF has a protective and restorative role on spermatogenesis after exposure to a high dose of alkylating agents through the stimulation of undifferentiated spermatogonia proliferation. Following G-CSF injections, mice exhibited better recovery of spermatogenesis after busulfan treatment compared to non-injected controls [55]. The mechanism of action involves G-CSF binding to the surface receptor (CSF3R) of undifferentiated spermatogonia and stimulation of spermatogenesis [56]. Therefore, given that various forms of G-CSF treatments are FDA approved, the clinical application in male fertility preservation during cancer treatments seems to be one step closer [56]. However, further clinical studies are needed in order to determine if G-CSF can effectively restore fertility after cytotoxic treatments.

### 5.3. Antioxidant Treatment

It is well-known that chemotherapeutic drugs increase oxidative stress in the testis by generating free reactive oxygen species (ROS) which can negatively influence spermatogenesis and impair sperm function, leading to increased risk of male infertility [57]. According to recent studies, melatonin has been proposed as a candidate to protect against germline toxicity induced by busulfan [58]. Busulfan, an alkylating gonadotoxic agent, administered in chronic myelogenous leukemia can damage the testicular function by different ways, including oxidative stress [59,60]. Studies in mice revealed that melatonin alleviates busulfan-induced toxicity through ROS elimination and apoptosis inhibition of spermatogonial progenitor cells. It has been proven that mouse spermatogonial progenitor cells express melatonin receptors suggesting that melatonin can act directly on these cells [61]. In addition, other studies indicate that melatonin does not interfere with the anti-tumoral effect of busulfan and hence, it can protect fertility without affecting the outcome of the oncological treatment [62,63]. In vivo studies in mice showed that only the combination of melatonin injection prior to chemotherapy and long-term injection during treatment was able to alleviate the toxicity of busulfan. Hence, one of the main limitations of using melatonin as a fertility-protective agent is its short half-life that reduces the efficiency of the treatment [61]. Moreover, given that the exact mechanism of melatonin action has not been elucidated yet, it creates concerns about undesirable side effects and should be further investigated.

According to a study of Carmely et al., (2009) the immunomodulatory AS101 can offer protection against cyclophosphamide-induced testicular damage in a mouse model [64]. Normally, AS101 is used in combination with chemotherapy to reduce side effects like neutropenia and hair loss without decreasing the anti-tumoral outcome of the therapy. Therefore, experimental approaches have shown that AS101 was able to protect against sperm DNA damage when it was co-administered with cyclophosphamide in mice. The mechanism of action is not clear, but it seems to act through Akt/GSK-3β pathway which has an important role in DNA repair and it is responsible for the improved chromatin structure. However, further studies are necessary to evaluate sperm quality and possible embryogenetic defects, reassuring the birth of healthy live offspring [64].

### 5.4. Future Approaches

The current preclinical approaches indicate that there is a fragile balance between ‘’killing’’ cancer cells and ‘’rescuing’’ germ cells which should be leaning towards the benefit of the patient. Therefore, better knowledge of the chemotherapy-induced testicular damage is critical to reveal the molecular mechanism of gonadotoxicity and design new fertility preservation strategies.

#### 5.4.1. MicroRNAs as Fertility Preservation Tools

Recently, an alternative therapeutic approach has been introduced based on miRNAs in the field of female fertility preservation, and which can be further developed to protect the testicular function from chemotherapy-induced toxicity [65,66,67]. According to the authors, miRNA replacement to restore the function of let-7a during exposure to 4-hydroxyperoxycyclophosphamide reduced the levels of apoptosis and hence protected the early developmental stage follicles from chemotherapy-induced toxicity. Additionally, the characterization of miRNAs in testicular function can contribute to identification of miRNAs with spermatogenesis-protective properties. miRNAs are short non-coding RNA molecules (~20–22 nucleotides) that are major post-transcriptional gene regulators as they can bind to mRNA targets by base pairing leading to mRNA cleavage or to translational inhibition [68]. These small regulators have great potential and their natural properties make them interesting molecules to be included in male fertility preservation strategies. According to this concept, miRNAs can be used to modulate the expression of genes involved in pathways that are activated in testis during oncological treatments such as cell arrest, DNA damage response and apoptosis [29,39] (Table 1).

**Figure 3:**
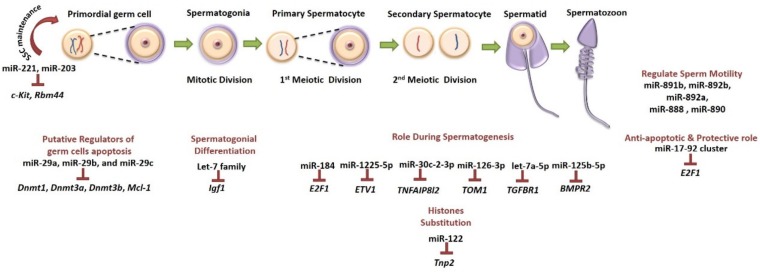
Schematic representation of selected miRNAs expressed during the several stages of development and maturation of male germ cells. Targeted genes and their functions are indicated. miR-221 and miR-203 contribute to SSC maintenance [83] while members of let-7 family regulate spermatogonial differentiation [84]. Several miRNAs (miR-122, miR-184, miR-1225-5p, miR-30c-2-3p, miR-126-3p, let-7a-5p and miR-125-5p) and their targets have been identified to have a critical role during spermatogenesis [85] and histone remodeling (miR-122) [86]. Moreover, miRNAs have been identified in germ cell apoptosis (miR-29 family) [79] and others have an anti-apoptotic role (miR-17-92) [77], whereas some miRNAs can modulate sperm characteristics like motility (miR-891b, miR-892b, miR-892a, miR-888 and miR-890) [87]. Inverted T bars indicate the targeted genes.

Several studies have proven that miRNAs play key roles in processes like growth, differentiation and cellular death, while their contribution in spermatogenesis and testicular function has been demonstrated [69] (Figure 3). Today, there are several databases available for miRNA-target prediction, providing essential information about gene networks and pathways controlled by miRNAs (TargetScan [70], TarBase [71], miRTarBase [72]). The regulation of different mRNAs by only one miRNA offers the opportunity of multiple and expanded targeting. One of the most important qualities of miRNAs is that they have tissue- and even cell-specific origin which enables the specific targeting of the tissue where they are expressed. Moreover, studies have shown that miRNA expression levels are influenced by cytotoxic agents like chemotherapeutic drugs [73] and a lot of them have been identified in tumoral chemoresistance [74]. In addition, there is evidence that miRNAs promote or inhibit cancer cells’ chemosensitivity [75] and therefore, they can be useful in minimizing off-target toxicity in the testis during oncological treatments. The identification of the miRNA(s) with fertility-protective properties can emerge as an innovative pharmacological option, but the feasibility of this idea is based on the use of an appropriate delivery system that will facilitate the transfer of the miRNA into the targeted cells of the testes. Currently, the field of nanotechnology has been significantly improved and next generation carriers have been developed. However, more research is required for improving targeted miRNA therapeutic approaches.

#### 5.4.2. Predictive Biomarkers of Chemotherapy-Induced Infertility

Recently, a study revealed that the mammalian-specific melanoma-associated antigen (MAGE) gene family is expressed during spermatogenesis and has a critical role in male germline protection against environmental stress caused by the lack of nutrients or genotoxic stress associated with chemotherapy. According to the authors, Mage-a KO mice presented increased levels of p53 in the germline which may sensitize these cells to genotoxic stress indicating that polymorphisms in human *Mage-a* genes can be related to increased risk of impaired spermatogenesis after chemotherapy [88]. Therefore, it can be hypothesized that *Mage-a* genes can be developed as biomarkers to predict the sensitivity of some patients to chemotherapeutic drugs, leading to a personalized counseling for fertility preservation before treatment initiation.

## 6. Experimental Strategies Based on Cryopreserved Immature Testicular Tissue

**Figure 4:**
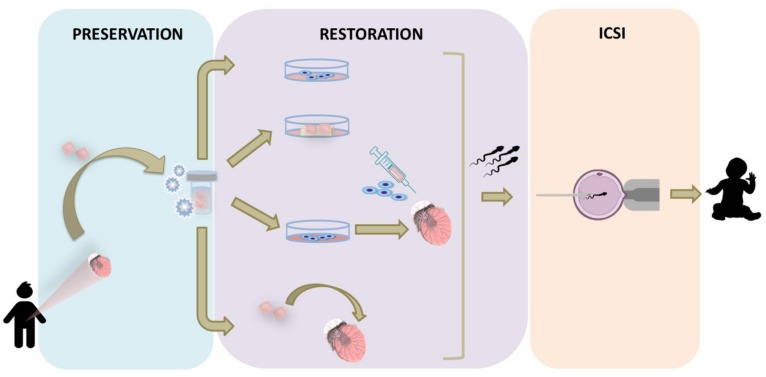
Overview of the experimental in vitro and in vivo strategies developed for boys based on the use of immature testicular tissue. Testicular tissue containing SSCs, obtained by biopsy before the initiation of the gonadotoxic treatment, can be cryopreserved and used later in life for fertility restoration. Differentiation of SSCs cultured either as single cells obtained after testicular tissue digestion, or in organ culture, can lead to sperm generation (in vitro approaches). Alternatively, SSCs can be transplanted following propagation into the rete testis of the patient, or intact testicular tissue can be orthotopically transplanted back to the individual (in vivo approaches). Sperm retrieved by any of the proposed strategies can be used for intracytoplasmic sperm injection (ICSI).

ITT banking is an option currently offered by a number of specialized medical centers to boys facing SSC loss. Although the first specimens of ITT were cryopreserved in 2002, there has been an increasing interest over the last few years with more than 700 patients [10,14] opting for testicular tissue cryopreservation for fertility restoration purposes worldwide. However, so far, there is no report of any of the proposed experimental approaches resulting in spermatozoa generation from human ITT (Figure 4).

### 6.1. In Vitro Approaches

In vitro spermatogenesis aims at culturing single cells isolated from ITT or intact ITT in order to obtain mature germ cells. In contrast to the in vivo approaches, this approach can be offered to all pediatric cancer survivors as it eliminates the risk of reintroducing malignant cells. The two-dimensional (2D) cell culture has been shown to support the differentiation of human meiotic germ cells into elongated spermatids and mature spermatozoa when co-cultured with Vero cells or Sertoli cells [89,90,91]. Although studies reported the differentiation of adult SSCs from cryptorchid and non-obstructive azoospermic patients to haploid round spermatids, they failed to demonstrate completion of the spermatogenic process [92,93,94]. However, the aforementioned studies were performed using adult testicular tissue and the contamination of mature germ cells in the starting suspension cannot be excluded.

The three-dimensional (3D) cell culture combines the culture of germ cells and somatic cells within 3D supportive matrices. This system appears to be more efficient for the in vitro germ cell development as it provides similar conditions to the in vivo situation enabling the cell-to-cell communication. The use of a 3D semi-solid culture system has been reported to support the differentiation of rat germ cells up to the post-meiotic stage and the formation of elongated spermatids in mice [95,96]. The low efficiency of this system though led to the generation and use of a scaffold that would mimic the testicular architecture. In 2017, Baert et al. described the culture of testicular cells on extracellular matrix obtained by decellularization of adult human testes [97]. The organoids supported germ cells for a period of four weeks, while their functionality was confirmed by immunofluorescence, hormonal analysis (T and inhibin B production) and specific cytokine secretion. Different approaches have been developed so far for the generation of testicular organoids. These include the three-layer Matrigel gradient system in rats supporting the formation of tubule-like structures with a functional blood–testis barrier and the survival of undifferentiated germ cells for up to 21 days [98]; the hanging drop culture method incorporating adult germ cells and immortalized Leydig and Sertoli cells supporting germ cell survival and T production [99]; and the microwell culture system tested with testicular cells from prepubertal pigs, mice, primates and humans exhibiting consistent testis-specific architecture after five days, with a well-defined interstitial compartment and seminiferous epithelium separated by a basement membrane [100].

However, the most successful system of in vitro spermatogenesis described so far is the organotypic culture. Culture of mouse ITT for three weeks resulted in mature spermatozoa production and the generation of fertile offspring by intracytoplasmic sperm injection (ICSI) [101,102,103]. Long-term organotypic culture of prepubertal human testicular tissue demonstrated the presence of intact seminiferous tubules and functional Sertoli and Leydig cells for up to 139 days [104]. Although, in this study, the maturation of human ITT and the functionality of the somatic cells were confirmed, differentiation of spermatogonia did not occur while there was a gradual loss of germ cells throughout the culture period. In a follow-up study of the same group, the use of different media containing factors involved in germ cell self-renewal and differentiation resulted in spermatogonia differentiation up to round spermatids after 16 days of culture [105]. Yet, the possibility for complete differentiation as well as the genetic and epigenetic normality of the generated germ cells require further investigations.

An interesting in vitro approach has been recently introduced by Baert et al., (2019) employing tissue engineering and 3D bioprinting [106]. The group reported the production of testicular constructs from 3D printed scaffolds with macropores seeded with testicular cell suspensions from prepubertal mice. Despite the fact that the tubular architecture was not restored, differentiation of immature germ cells up to the stage of elongated spermatids was achieved. Similarly, but in the female reproductive field, Laronda et al. (2017) demonstrated bioprinted scaffolds supporting the growth of mouse follicles [107]. Additionally, the transplantation of the bioprosthetic ovaries in sterilized mice resulted in highly vascularized scaffolds, restoration of the ovarian function and offspring generation through natural mating. Both studies provide encouraging results for future application of 3D bioprinting for fertility restoration.

### 6.2. In Vivo Approaches

#### 6.2.1. Spermatogonial Stem Cell Transplantation

Autotransplantation of SSCs is a fertility restoration option that involves the injection of SSCs, isolated from cryopreserved ITT, into germ cell ablated testis. The technique was successfully introduced in 1994, when mouse testicular cells containing SSCs were injected into germ cell depleted seminiferous tubules resulting in the colonization of the recipient’s empty niches and establishment of donor-derived spermatogenesis [108,109]. The generation of mouse offspring from the transplanted SSC-derived spermatozoa after ICSI [110] and natural mating [111] confirmed the feasibility of the technique. Since then, SSCs transplantation was performed in several species, with either fresh or cryopreserved SSCs, resulting in complete spermatogenesis [112,113]. The potential clinical implementation of the technique was demonstrated by a study from Hermann et al. (2012) describing autologous SSC transplantation in a non-human primate [114]. In that study, frozen–thawed SSCs were injected into the testes of rhesus monkeys, that were rendered infertile with alkylating chemotherapy, resulting in spermatozoa generation, oocyte fertilization via ICSI and blastocyst formation. In humans, there is only one report of adult SSC autotransplantation by injection in the rete testis of seven patients following treatment for non-Hodgkin’s lymphoma [115]. However, the outcome of this study was never published.

The technique is limited by the low number of SSCs in the germ cell population, estimated to represent only 0.02%–0.03% of germ cells in the mouse [116] and 3.5% in non-human primates [117], and the low colonization efficiency since only 12% of transplanted SSCs are able to colonize the recipient’s niche [118]. Therefore, efficient protocols for in vitro propagation of SSCs are required [119,120,121]. Interestingly, a study from Kadam et al. (2018) demonstrated an increased efficiency when SSCs were co-transplanted with mesenchymal stem cells previously treated with transforming growth factor b1 (TGFb1) [122].

A number of safety concerns arise with SSC transplantation from cryopreserved ITT. The epigenetic and genetic safety are essential; however, studies addressing these issues are currently limited in the field of reproductive medicine. In a study of Goossens et al., (2010) no alterations in the genome of mouse offspring born after SSC transplantation were observed [123] and normal expression pattern of epigenetic markers (DNA methyltransferase 3A and histone 4 lysine 5 acetylation) was reported in mice obtained after co-transplantation of SSCs and TGFb1-treated mesenchymal stem cells [124].

Additionally, following SSC propagation and transplantation, the incidence of cancer and the life span of mice were similar between the transplanted and control groups [125]. However, the risk of reintroducing cancer cells in patients diagnosed with metastatic or hematological malignancies is high. Twenty transplanted leukemic cells were found to be enough to induce cancer relapse in rats [126]. Although de-contamination of human testicular cell suspensions from cancerous cells by cell-sorting techniques has been studied, the results were not encouraging [127,128,129]. However, a different decontamination approach described by Sadri-Ardekani et al. (2014) demonstrated the elimination of acute lymphoblastic leukemia cells from human SSC propagation culture after 26 days [130].

#### 6.2.2. Testicular Tissue Transplantation

Autotransplantation of ITT, an alternative fertility restoration strategy, involves the transplantation of SSCs within their intact microenvironment. The feasibility of immature human gonadal tissue transplantation has already been proven by successful transplantation of cryopreserved ovarian tissue [131] and while it is still considered an experimental procedure more than 90 babies have been born so far [132]. Developmental differences between the male and female gonad render ITT transplantation challenging, but at the moment it appears more promising than the aforementioned strategies.

Successful restoration of ITT functionality following transplantation and establishment of spermatogenesis with generation of offspring has been reported in several species [133,134,135]. However, the studies utilizing ITT from non-human primates are of a higher significance due to their closer phylogenetic distance to humans. Autotransplantation of fresh marmoset ITT [136] as well as the transplantation of cryopreserved ITT from rhesus monkey [137] into the scrotum resulted in complete spermatogenesis. In the latter study, only 5% of the transplants were recovered and the efficiency of spermatozoa production was low, highlighting the impact of the cryopreservation and thawing protocols. An important milestone for the clinical translation of ITT transplantation was the first birth of a non-human primate from sperm generated following ITT autotransplantation [138]. In this study, both fresh and frozen ITT from rhesus monkeys was transplanted under the back skin and into the scrotum of castrated males. Following a transplantation period of 8 to 12 months, the recovery rate of testicular transplants was 100% and complete spermatogenesis was confirmed in all transplants. Spermatozoa retrieved from frozen–thawed scrotal transplants were used to fertilize 138 oocytes by ICSI, resulting in the generation of 39 blastocysts. A total of 11 blastocysts were transferred into six females resulting in a pregnancy and the birth of “Grady”. It remains questionable though if the initial treatment that the recipients had with busulfan for another experiment and which failed to eradicate spermatogenesis was stronger, could possibly have influenced transplant survival and spermatogenesis. Chemotherapy and irradiation can induce damage to the vasculature, especially to small vessels supporting the walls of large vessels [139]. Yet, strategies to improve revascularization of ITT transplants by administration of angiogenesis-supporting factors, such as vascular endothelial growth factor, have been reported with positive results [140,141].

Autotransplantation of human ITT has not yet been reported. The studies conducted so far with human ITT involve the xenotransplantation of the tissue in immunodeficient nude mice [141,142,143,144,145,146]. Despite the success of ITT xenotransplantation in other species, human transplants exhibit low spermatogonial survival and reduced spermatogonial differentiation. Even though the post-transplantation period varies from 4 up to 12 months, complete spermatogenesis with the generation of haploid gametes has not been reported besides the differentiation of the spermatogonia up to the spermatocyte stage. As it has been hypothesized by Ntemou et al., (2019) maturation of testicular tissue and spermatozoa formation in human ITT transplants may require a more prolonged post-transplantation period [141,147]. Nonetheless, xenotransplantation of human ITT should only be considered as an experimental method for identifying factors and improving conditions which may influence the outcome of the procedure before it can be implemented into a clinical setting.

#### 6.2.3. Clinical Challenges and Concerns on ITT Transplantation

Whilst planning the first clinical trial on human ITT transplantation a number of questions need to be addressed and taken into consideration. Initially, and in order to ensure its safety, ITT transplantation should be offered only to patients diagnosed with solid tumors, or non-malignant hematological conditions receiving preconditioning therapy. For patients with metastatic or hematological cancer, such as leukemia, it is however not recommended as the risk of the collected testicular tissue being contaminated with malignant cells causing a disease relapse cannot be discarded.

In addition, defining the optimal time as well as the optimal site for ITT transplantation is critical. In the recent study of Fayomi et al., (2019) the tissue was transplanted back to the recipients during prepuberty which ensured the natural maturation of the tissue and establishment of spermatogenesis while the monkeys entered puberty [138]. However, in a clinical situation the option for ITT transplantation during adulthood appears to be more reasonable. First, it is possible that spontaneous recovery of spermatogenesis may occur even years after the gonadotoxic treatment [35] and therefore ITT transplantation will not be required and second, the time a transplant remains functional post-transplantation is unknown. ITT can be transplanted either to an ectopic or to an orthotopic location. For most of the species, ITT transplantation both under the back skin and under the scrotal skin resulted in spermatozoa generation. However, for other species like the common marmoset and possibly humans, the transplantation site can be critical [136,147]. Initiation and maintenance of spermatogenesis in ITT depend on the temperature and hormonal environment [133], requiring higher T concentration than the normal serum levels [148]. The transplantation of ITT under the tunica albuginea and into the testicular parenchyma (intratesticular transplantation) appears promising [147] as it fulfils both conditions of the optimal temperature and T levels 25- to 125-fold higher within the testes than in the peripheral circulation [149].

The next step following ITT transplantation is the recovery of the transplants from the testicular parenchyma or scrotum to isolate spermatozoa which will be used for the generation of offspring through assisted reproduction techniques. Although the process of spermatogenesis under physiological conditions requires approximately 74 days [150], the time required for the maturation of human ITT is still unknown. In addition, with the presence of fluid in the lumen of the seminiferous tubules and its constant accumulation there is a risk of seminiferous epithelium damage [134] minimizing further the optimal time window for spermatozoa retrieval. In order to identify the optimal time for spermatozoa retrieval, a regular follow-up of the patients and hormonal analysis (T, FSH, inhibin B, insulin-like 3) that will ensure the maturation and functionality of the transplanted tissue as well as the production of anti-sperm antibodies revealing the presence of spermatozoa will be required.

Since spermatozoa have not yet been obtained following human ITT transplantation, their fertilization capacity remains questionable. Nonetheless, Honaramooz et al. (2004) reported a 19% blastocyst rate with spermatozoa generated in ectopic xenotransplants from rhesus monkey [151]. More recent studies demonstrated the competence of transplant-derived sperm to fertilize oocytes leading to pregnancy and live birth of offspring from cynomolgus monkey (xenotransplants) [152] and rhesus monkey (autotransplants) [138] implicating the feasibility of the technique for fertility restoration.

Despite the birth of offspring from transplant-derived sperm, studies assessing the genetic and epigenetic integrity of spermatozoa are lacking. Only one study from Goossens et al., (2011) designed to characterize epigenetic modifications following ITT transplantation, reported no differences in the expression levels of enzymes catalyzing DNA methylation, the DNA methylation status and stage-specific histone modifications [153]. However, this study was conducted with fresh tissue while the cryopreservation procedure could influence the outcome. It is encouraging though that the non-human primate offspring born so far are characterized as “normal” based on developmental and behavioral tests [138,152].

## 7. Conclusions

With the growing population of childhood cancer survivors, there is an urgent need to develop strategies to safeguard the fertility of this group of patients. The aforementioned studies mostly describe experimental approaches that could become future routine strategies for male fertility management, aiming at preventing, protecting and restoring the testicular function against oncological treatments. In the first instance, the development of novel approaches protecting germ cells and somatic cells during treatment, regardless of the cancer diagnosis, would potentially diminish the need for the invasive ITT collection and cryopreservation. However, recent advances based on the use of ITT and mostly the in vivo approaches (i.e., ITT transplantation) are encouraging but limited to certain cancer patients. Additionally, before any clinical implementation, validation of the safety is essential for the decision-making process as well as adequate counseling of the patients.

## Figures and Tables

**Table 1:** miRNAs implicated in testicular function and germline apoptosis.

miRNAs in Male Germline Apoptosis Regulation				
miRNA	Targeted Genes	Function	Species	Reference
miR-16	*Ccnd1*	Apoptosis induction	pig	[76]
miR-17-92	*c-MYC, E2F1*	Downregulation leads to testicular atrophy, apoptosis induction and germ cell free seminiferous tubules	mouse	[77,78]
miR-29	*Dnmt, Mcl-1*	Extensive germ cells apoptosis	rat	[79]
miR-122	Unknown	Inhibition minimizes ochratoxin-A-toxicity	spermatocyte-like cell line	[80]
miR-144	*FASL, CAS3, TP53, BCL2L1*	Regulates apoptosis-related genes, apoptosis induction	sheep	[81]
miR-449, miR-34b/c	E2F-pRb pathway	Acts redundantly to suppress E2F-pRb pathway during the meiotic phase of spermatogenesis	mouse	[82]
miR-34	Genes involved in cell cycle, apoptosis and growth factor signaling	Regulates cell cycle spermatogenesis progression and sperm senescence	pig	[76]

## Abbreviations

AMH	anti-Müllerian hormone
AR	androgen receptor
FSH	follicle stimulating hormone
G-CSF	granulocyte colony-stimulating factor
GnRH	gonadotrophin-releasing hormone
GnRHa	gonadotrophin-releasing hormone agonist
ICSI	intracytoplasmic sperm injection
ITT	immature testicular tissue
LH	luteinizing hormone
MAGE	melanoma-associated antigen
miRNA	microRNA
ROS	reactive oxygen species
SSC	spermatogonial stem cell
TTGFb1	testosteronetransforming growth factor b1
